# Chinese Skullcap in Move Free Arthritis Supplement Causes Drug Induced Liver Injury and Pulmonary Infiltrates

**DOI:** 10.1155/2013/965092

**Published:** 2013-04-14

**Authors:** Renumathy Dhanasekaran, Victoria Owens, William Sanchez

**Affiliations:** ^1^Division of Gastroenterology and Hepatology, Mayo Clinic, Rochester, MN 55905, USA; ^2^Department of Pathology, Mayo Clinic, Rochester, MN 55905, USA

## Abstract

Herbal medications are being increasingly used by the American population especially for common conditions like arthritis. They have been reported to cause adverse effects, including significant hepatotoxicity, but reporting remains sporadic. We report here a patient who developed drug induced liver injury following the intake of Move Free, which is an over-the-counter arthritis supplement. We propose that Chinese skullcap, which is one of the herbal ingredients of the medication, is responsible for the adverse event. There was a strong temporal association between the intake of supplement and onset of symptoms, and also there have been a few recent case reports implicating the same component. A unique observation in our case is the occurrence of pulmonary infiltrates simultaneously with the hepatotoxicity, and this side effect has not been well documented before. Both the hepatic and pulmonary complications completely resolved over few weeks after the patient stopped taking the medication. Since these supplements are readily available over the counter, we feel that it is important to document possible adverse outcomes to raise awareness in the medical community and also among patients.

## 1. Introduction

More than a third of Americans use herbal medications for various purported beneficial effects and most of them do not disclose their use to physicians [1, 2]. These medications are considered by patients to be safe as they are of natural origin. Unfortunately they can cause serious adverse effects. The process of FDA regulation of these supplements is less rigorous than that for drugs, so their adverse effects are not well documented. Nonetheless it is important to document cases of serious adverse outcomes related to herbal supplements, both to raise awareness and hopefully to prevent recurrence. We report here a case of hepatotoxicity related to use of an over-the-counter arthritis supplement.

## 2. Case Report

**Figure 1:**
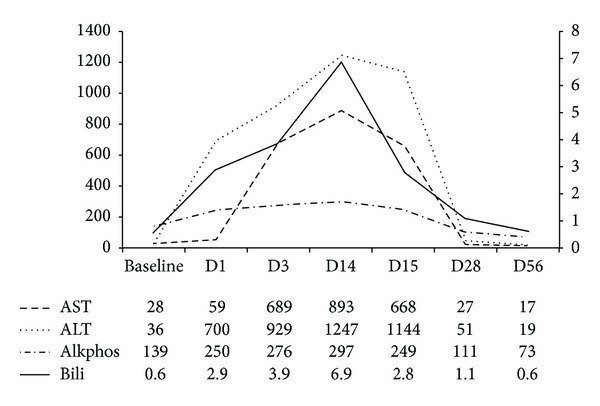
The graph depicts the rise and fall of transaminases and bilirubin from the time of 8 weeks after presentation. The table below the graph shows the numerical values. (*Y*-axis on left side is in IU scale and *Y* axis on right side is in mg/dL scale).

A 62-year-old Caucasian female with a history of diabetes mellitus was hospitalized for acute onset of shortness of breath. She was found to be hypoxic, and chest X-ray showed bilateral interstitial infiltrates. She was incidentally found to have abnormal liver tests in the form of elevated aminotransferases and hyperbilirubinemia (Figure 1). The patient did not give a history of prior liver disease, did not drink alcohol, and had no risk factors for acute viral hepatitis. Her most recent previous liver tests (from 3 years ago) had been completely normal. Physical examination revealed jaundice but no splenomegaly, asterixis, or other stigmata of chronic liver disease. The following tests were negative or normal on evaluation—viral hepatitis serologies, serologic markers of autoimmune hepatitis, serology for Lyme disease, HIV, CMV, and EBV serologies. Serum ceruloplasmin was normal, and testing for alpha1 antitrypsin deficiency was negative. Ultrasound of the abdomen demonstrated fatty infiltration with no evidence of portal hypertension and no evidence of biliary ductal obstruction.

A detailed medication history was obtained to identify potentially hepatotoxic medications. The patient reported using metformin at a stable dose on a daily basis for several years. Two weeks prior to hospitalization, she had started taking a new over-the-counter, multiingredient arthritis supplement called Move Free. She was using the product as directed, taking four tablets per day for two and a half weeks and had tapered down to two tabs a day for four days prior to hospitalization. The medication was noted to contain glucosamine, chondroitin, MSM (methylsulfonylmethane), Chinese Skullcap, black catechu, and maltodextrin. The herbal supplement, Chinese Skullcap has been implicated in causing drug induced liver injury in other cases.

**Figure 2:**
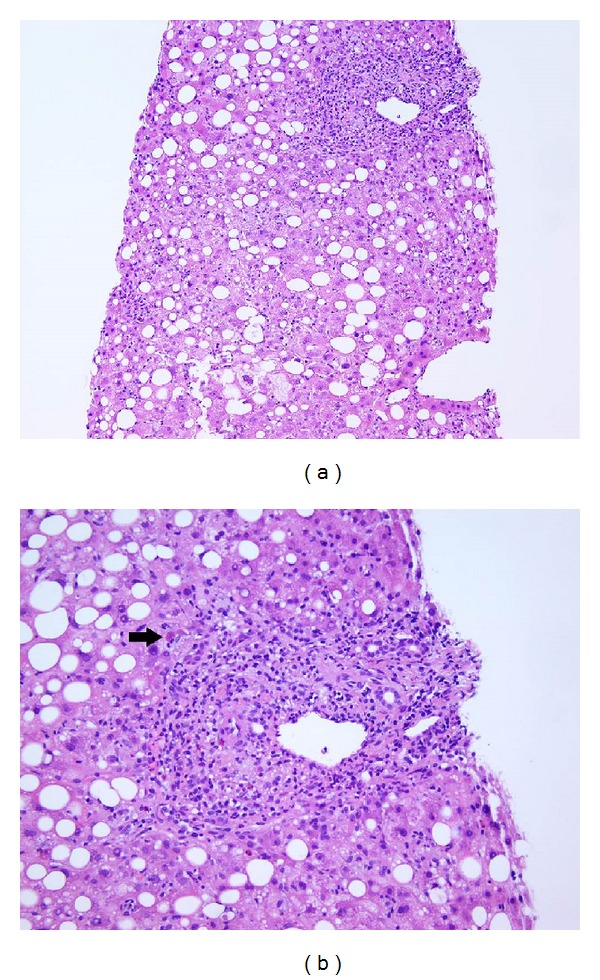
Low magnification of the liver biopsy showing histologic features of a panacinar hepatitis with moderate mixed portal and lobular inflammation ((a) Hematoxylin and Eosin, 100x). Higher magnification of the mixed portal inflammation with lymphocytes, plasma cells, and eosinophils with foci of lobular injury with an acidophil body (arrow) ((b) Hematoxylin and Eosin, 200x).

A liver biopsy (Figures 2(a) and 2(b)) was performed which showed histologic features of a panacinar hepatitis, with expansion of the portal tracts by a mixed chronic inflammatory infiltrate composed of lymphocytes, eosinophils, and plasma cells. The lobular parenchyma showed foci of inflammation with scattered eosinophils and acidophil bodies. There was mild steatosis (15% to 20%) with features of a mild superimposed steatohepatitis present without established fibrosis. The combination of clinical history, negative serologies, and biopsy findings strongly favored adverse drug reaction.

At initial presentation, the patient did have significant respiratory distress and required oxygen support. She did not have any fever or cough. Computed tomography (CT) of the chest revealed bilateral ground glass opacities in the upper lobes. Viral and bacterial testing failed to identify an infections etiology to her pulmonary infiltrates. It is possible that her pulmonary manifestations were also related to the Chinese skullcap. 

The patient discontinued the supplement and her hepatitis slowly improved over the next several weeks. In 2 weeks her AST was down to 668 from 893 and ALT was down to 1144 from 1247. After 8 weeks the liver enzymes had completely normalized (Figure 1). She required home oxygen for a few weeks after discharge and was eventually able to discontinue this. 

## 3. Discussion

Alternative and complementary medicines are being increasingly embraced by the US population. The CDC reports that 38% of American adults used complementary medicine in 2007 compared to 36% in 2002 [1]. Not surprisingly, arthritis related supplements were one of the more commonly used categories, with glucosamine being the second most commonly used supplement [1]. The manufacturers of herbal products are not mandated to acquire FDA approval for marketing, so their adverse effect profile is not well known. There are several case reports of adverse drug reactions (ADR) which have been attributed to different herbal products but causality is difficult to establish. Ma-huang, usnic acid, germander and kava are a few such herbal supplements which have been associated with severe liver injury [3–6].

Chinese skullcap (*Scutellaria baicalensis*) is an herb which is native to China and parts of Russia. Baicalein and baicalin are the two major flavonoids found in this herb and they have been reported to have anticancer and anti-inflammatory properties [7, 8]. A combined extract containing primarily baicalin from *Scutellaria baicalensis* and catechin from *Acacia catechu* was shown to inhibit COX-1 and COX-2 pathways, and hence they are used in joint supplements to relieve arthritis related pain [9]. Move Free is one such product advertised to promote joint health and slow cartilage loss, and its main ingredients are glucosamine, chondroitin, MSM (methylsulfonylmethane), Chinese skullcap, black catechu, and maltodextrin. 

The patient, we reported above, was diagnosed with hepatotoxicity most likely to be related to the use of Move Free herbal supplement. We used the Naranjo nomogram to assess whether the hepatotoxicity can be attributed to the Chinese skullcap ingredient [10]. The association falls under the “probable ADR” category with a score of 7. In our patient, the onset of the liver test abnormalities were closely temporally related to use of the medication and the liver tests normalized after the patient went off the medication. We did rule out most other alternate etiologies for the liver test abnormalities and the biopsy confirmed features of drug induced liver injury. In literature review, we also found previous conclusive reports of similar reaction to the Chinese skullcap. Moreover, the other components of the supplement such as glucosamine, chondroitin, and black catechu have not been associated with hepatotoxicity.

Prior case reports describe hepatotoxicity related to herbal supplements which contained skullcap as one of the components, but causality was not established [11–13]. There have been three recent case reports of hepatotoxicity specifically related to Chinese skullcap found in Move Free supplement [14, 15]. The first paper in 2010 by Linnebur et al. reported two cases of hepatotoxicity in patients who took this arthritis supplement [14]. Both of them were women older than 70 years and the liver test abnormalities were found 3 weeks after onset of taking Move Free Advanced, which is similar to our patient. But neither of these patients had a liver biopsy. The third case report in 2012 was also an older woman in her 70s who developed liver test abnormalities within 2 weeks of starting this supplement, and this reaction recurred when she rechallenged herself with Move Free Advanced [15]. Her liver biopsy showed changes suggestive of drug induced liver injury. Neither of these case reports mentions any pulmonary complications. 

Pulmonary and hepatic complications related to taking herbal supplements containing skullcap (*Scutellaria galericulata*) and Comfrey (*Symphytum officinale*) have been reported in one patient in a letter to the editor in 1992 [16]. This patient had developed bilateral reticulonodular changes on chest radiograph along with abnormal liver tests, similar to our patient. Both the liver tests and chest radiograph had improved several weeks after discontinuing the herbal supplement. The etiology of pulmonary complications was attributed to Comfrey and not skullcap, as Comfrey had been reported to cause pulmonary complications from endothelial dysfunction. We conducted a literature search to see if any of the other ingredients of Move Free such as chondroitin, glucosamine, MSM, or black catechu has been associated with pulmonary complications; we did not find any such association. Given the strong temporal association and negative infectious workup, it is possible that the pulmonary infiltrates and hypoxia were also related to the Chinese skullcap. 

In conclusion, our case adds to the literature which reports severe hepatotoxicity related to the Chinese skullcap ingredient of Move Free arthritis supplement. And this is the first case report to propose that this supplement can also potentially cause pulmonary complications. These over-the-counter medications are readily available with no mention of potential serious side effects on their label. Hence we believe that reporting these side effects in the literature is important to raise awareness among both patients and physicians.
